# Supplementary material for: Clinicopathological Features of Non-Small Cell Lung Carcinoma with NRAS Mutation
Source: J Pers Med. 2025 May 16;15(5):199. doi: 10.3390/jpm15050199 (PMC12113192; doi:10.3390/jpm15050199)
Supplement: Supplementary file 1 [file jpm-15-00199-s001.zip › jpm-3533599-supplementary.pdf]

Case N.	Sex	Age	Exon	Codon	Smoking status	Therapy	PD-L1
1	M	63	3	61			
2	M	58	3	13	Ex smoker	immunotherapy (atezolizumab). then carboplatin and paclitaxel	
3	F	72	3	61	Ex heavy smoker	pemetrexed	low
4	M	63	2	12			low
5	F	66	3	61	Smoker 10 cig/day from 14 years old.		neg
6	F	83	2	12			low
7	M	46	3	61			high
8	F	81	4	142			low
9	F	76	3	61		RT	low
10	M	51	3	61			
11	M	79	3	61		RT. imatinib	neg
12	M	65	3	61			low
13	M	71	3	61			low

Supplementary Table S1. Additional clinical data. Legend: cig cigarettes. RT radiotherapy

NRAS Mutations (1: 61 vs 2: non-61)		Time	Status	Cumulative Proportion Surviving at the Time	
				Estimate	*Std. Error*
1	1	1.000	1	0.875	0.117
	2	3.000	1	0.750	0.153
	3	4.000	0	.	.
	4	24.000	0	.	.
	5	27.000	1	0.563	0.199
	6	35.000	0	.	.
	7	41.000	0	.	.
	8	48.000	0	.	.
2	1	0.000	1	0.667	0.272
	2	12.000	1	0.333	0.272
	3	14.000	1	0.000	0.000

Supplementary Table S2. Survival Table. Legend: Std. standard

NRAS Mutations (1: 61 vs 2: non-61)	Mean[a]				Median
	Estimate	Std. Error	95% Confidence Interval		Estimate
			Lower Bound	*Upper Bound*	
1	32.563	7.112	18.623	46.502	.
2	8.667	4.372	0.098	17.235	12.000
Overall	25.273	6.231	13.060	37.485	27.000

Supplementary Table S3. Means and Medians for Survival Time. Legend: Std. standard; a) estimation is limited to the largest survival time if it is censored.
